# Pathological and Virological Studies of p16-Positive Oropharyngeal Carcinoma with a Good Response to Neoadjuvant Chemotherapy

**DOI:** 10.3390/microorganisms8101497

**Published:** 2020-09-29

**Authors:** Daisuke Inukai, Taichi Kan, Shunpei Yamanaka, Hiroki Okamoto, Yasushi Fujimoto, Takanori Ito, Natsuki Taniguchi, Yuuki Yamamoto, Toyonori Tsuzuki, Akiyoshi Takami, Tetsuya Ogawa

**Affiliations:** 1 Department of Otolaryngology, Aichi Medical University, Nagakute, Aichi 480-1195, Japan; inukai.daisuke.006@mail.aichi-med-u.ac.jp (D.I.); kan.taichi.034@mail.aichi-med-u.ac.jp (T.K.); yamanaka.shunpei.036@mail.aichi-med-u.ac.jp (S.Y.); okamoto.hiroki.251@mail.aichi-med-u.ac.jp (H.O.); fujimoto.yasushi.839@mail.aichi-med-u.ac.jp (Y.F.); 2 Department of Surgical Pathology, Aichi Medical University Hospital, Nagakute, Aichi 480-1195, Japan; itou.takanori.010@mail.aichi-med-u.ac.jp (T.I.); taniguchi.natsuki.086@mail.aichi-med-u.ac.jp (N.T.); yamamoto.yuuki.087@mail.aichi-med-u.ac.jp (Y.Y.); tsuzuki.toyonori.046@mail.aichi-med-u.ac.jp (T.T.); 3 Department of Internal Medicine, Division of Hematology, Aichi Medical University, Nagakute, Aichi 480-1195, Japan; takami-knz@umin.ac.jp

**Keywords:** p16-positive oropharyngeal carcinoma, neoadjuvant chemotherapy, pathological complete remission

## Abstract

Human papillomavirus (HPV)-related, p16-positive oropharyngeal carcinoma is considered to be sensitive to anticancer drugs, and the standard treatment is therefore chemoradiotherapy, rather than surgery, especially for aggressive disease. However, with this higher sensitivity, chemotherapy alone may achieve a pathological complete response (CR), making radiation therapy unnecessary. A 46-year-old man with p16-positive squamous cell carcinoma (SCC) of the lateral oropharynx (palatine tonsil) underwent neoadjuvant chemotherapy. This achieved clinically significant tumor shrinkage and therefore surgery was performed for subsequent definitive treatment. Clinical and CT findings indicated a good effect of neoadjuvant chemotherapy on the tumor. A biopsy prior to chemotherapy revealed SCC, which demonstrated p16 immunoreactivity and positive signals for high-risk HPV by RNA in situ hybridization. The post-chemotherapy surgical specimen showed pathological CR and no p16 positive cells nor positive signals for high-risk HPV those were detected in the pre-chemotherapy specimen. There are some reports of chemotherapy alone achieving pathological CR in cases of p16-positive oropharyngeal carcinoma, but none have included high-risk HPV RNA findings. This is the first report of the disappearance of cancer cells as well as p16 staining and a positive signal for high-risk HPV. Achieving pathological CR confirmed by immunohistochemistry and high-risk HPV RNA in situ hybridization in a solid tumor with chemotherapy alone suggests that chemotherapy may have both an antitumor effect and an antiviral effect. Forgoing subsequent radiotherapy and undergoing surgery might be unnecessary and follow-up instead might be sufficient in such cases. Into the future, in an optimal tailored treatment approach, the option of neoadjuvant chemotherapy should be considered for management of p16-positive oropharyngeal carcinoma. Other options such as tumor immunotherapy are also expected to be effective.

## 1. Introduction

Human papillomavirus (HPV)-related, p16-positive oropharyngeal carcinoma is sensitive to chemotherapy and radiotherapy [1,2,3], and show better prognosis than p16-negative oropharyngeal carcinoma. In recent years, p16 expression has reflected the American Joint committee on Cancer/ Union for International Cancer Control (AJCC/UICC) stage of the disease [4]. Different treatment strategies based on p16 expression have been proposed, and multiple clinical trials are underway to establish evidence for the effectiveness of such strategies [5].

p16-positive oropharyngeal carcinoma shows high chemosensitivity status and therefore medical oncologists view chemoradiation as the standard treatment rather than definitive surgical treatment [6]. However, with this higher sensitivity to anticancer drugs, it may be possible to achieve a pathological complete response (CR) by neoadjuvant chemotherapy alone.

Guidelines recommend lowering radiation and chemotherapy doses for patients with p16-positive oropharyngeal cancer, even in highly curable cases. Chemoradiation can cause a number of serious problems for patients, including feeding tube dependence for dysphagia, cerebrovascular disease due to carotid stenosis, xerostomia due to salivary gland dysfunction, and aesthetic problems due to lymphedema. [7,8]. Furthermore, with conventional radiation doses for curative intent, re-irradiating the same site or adjacent areas can be fatal [9]. Thus, p16-positive oropharyngeal carcinoma can be cured by only neoadjuvant chemotherapy, instead of chemoradiation therapy.

From the perspective of head and neck surgical oncologists, precision surgery can better preserve function than high intensity chemoradiation because the latter can adversely affect the normal mucous membranes and other parts of the body, which can impair swallowing and vocalization. In addition, in some cases, anticancer treatment before surgery, so-called neoadjuvant chemotherapy, may improve the disease status such that surgery is more likely to preserve function than radiation therapy. The significance of neoadjuvant chemotherapy includes prevention of distant metastasis, downstaging, before surgical treatment is performed [10,11]. There are also reports of surgery being curative for patients whose tumors shrank with chemotherapy and whose postoperative function could be preserved [12].

Here we report our experience treating a case of p16-positive squamous cell carcinoma (SCC) of the lateral oropharynx (palatine tonsil) in which neoadjuvant chemotherapy achieved clinically significant tumor shrinkage, which then allowed surgery to be performed as definitive treatment instead of radiotherapy. We report the interesting pathological findings by p16 immunostaining and RNA in situ hybridization for high-risk HPV genotypes before and after neoadjuvant chemotherapy.

## 2. Case and Methods

A 46-year-old man was admitted with swelling of the left palatine tonsil and tonsil biopsy showed SCC. Immunostaining for p16 was positive. Computed tomography (CT), magnetic resonance imaging (MRI), and positron emission tomography (PET-CT) showed a left tonsil tumor and cervical lymph node metastasis, but no obvious distant metastasis. A biopsy from the tumor was performed, and revealed squamous cell carcinoma with p16 expression. The diagnosis was clinical T2N1M0 (Stage I) oropharyngeal cancer with p16-positive.

**Figure 1:**
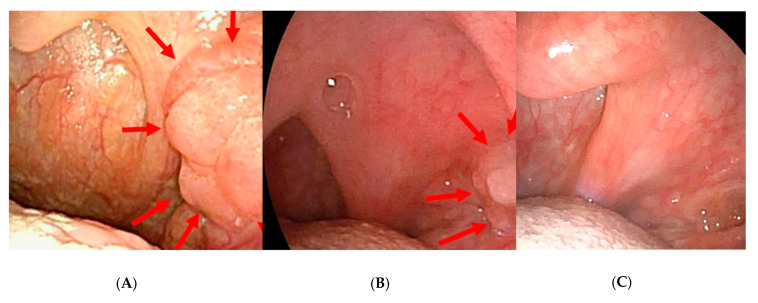
Gross findings of the primary lesion before and after neoadjuvant chemotherapy. (**A**) Left middle pharyngeal lateral wall (palatine tonsil) before chemotherapy showing a neoplastic lesion with papillary growth centered at the upper to middle pole (arrows). (**B**) Left oropharyngeal lateral wall after the first chemotherapy session showing a marked reduction in tumor size. (**C**) Left nasopharyngeal lateral wall after the second chemotherapy session showing disappearance of the tumor.

Before chemotherapy, the massive tumor in the left tonsil showed neoplastic changes with papillary-like growths mainly at the superior to middle pole (Figure 1A). After the first chemotherapy session, there was a marked reduction in size (Figure 1B), and the tumor was no longer evident macroscopically after the second course (Figure 1C). Our oncology team determined that the patient had achieved at least a partial response.

After the patient provided informed consent, neoadjuvant chemotherapy with cisplatin 70 mg/m^2^ was administered on day 1 and combination chemotherapy with fluorouracil (5-FU) 1000 mg/m^2^ was administered on days 1 to 5. The response was then checked by macroscopy, CT, and MRI. The treatment plan was to perform function-preserving surgery if the tumor had good response, but if there was no change in tumor status, subsequent radical treatment would be with done high-intensity chemoradiotherapy.

This case report was conducted in accordance with the following code of ethics. The ethics committee of Aichi Medical University’s ethics committee approval code for this study is 2016-H71, and the use of personal data was designed to ensure that no individuals could be identified. Descriptions of medical history and patient background were omitted in the text, and written informed consent from the patient was deemed unnecessary.

## 3. Results

### 3.1. Macroscopic Findings of the Primary Lesion before and after Neoadjuvant Chemotherapy

Before chemotherapy, the massive tumor in the left tonsil showed neoplastic changes with tumor-like growth compared to the contralateral tonsil mainly at the superior to middle pole (Figure 1A). After the first chemotherapy session, there was a marked reduction in size (Figure 1B), and the tumor was no longer evident macroscopically after the second course (Figure 1C). Our oncology team determined that the patient had achieved at least a partial response.

### 3.2. CT image Findings before and after Neoadjuvant Chemotherapy

**Figure 2:**
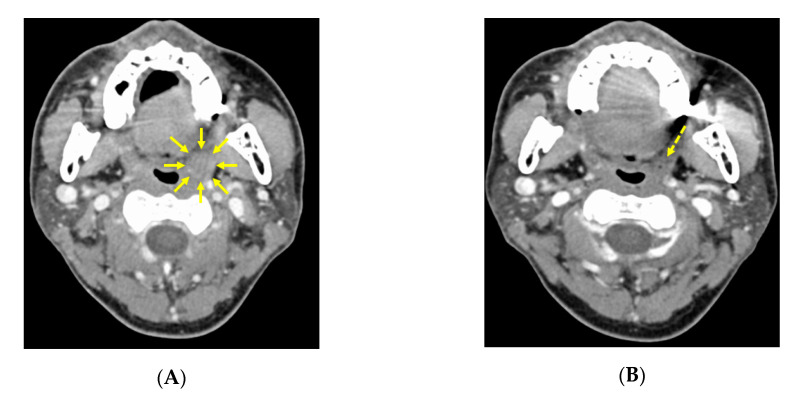
CT imaging findings of the primary tumor before and after neoadjuvant chemotherapy. (**A**) Pre-chemotherapy CT scan showing the left oropharyngeal lateral wall with the primary tumor is larger than the right side. The tumor is about 15 × 15 mm (arrows). (**B**) CT scan after the second neoadjuvant chemotherapy course showing a markedly smaller remnant left tonsil mass.

**Figure 3:**
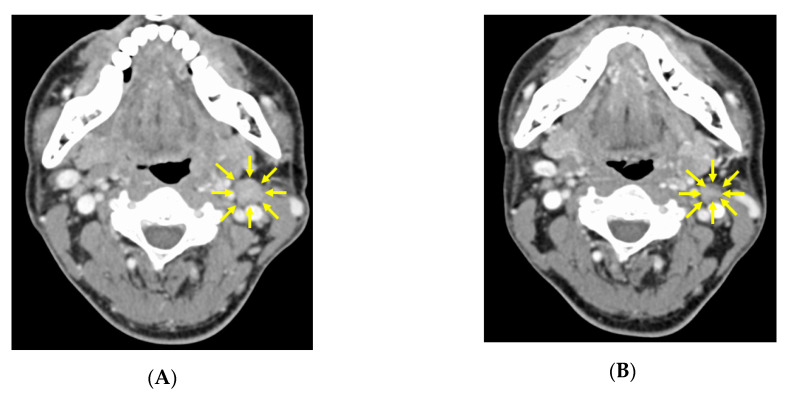
CT imaging findings of the left cervical lymph node region before and after neoadjuvant chemotherapy. (**A**) Before neoadjuvant chemotherapy, a 12 × 13 mm swollen lymph node is evident in the left upper internal deep cervical lymph node region (arrows). (**B**) After the second neoadjuvant chemotherapy course, the enlarged lymph node has shrunk to 10 × 10 mm (arrows).

Before chemotherapy, CT findings showed the left tonsil was 15 × 15 mm larger than the right tonsil (Figure 2A). After the second course, the tumor had shrunk markedly, and the borderline between the tumor and the normal tonsil became unclear, with the size of the tumor being up to 5 mm at most (Figure 2B). Also before chemotherapy, a 12 × 13 mm lymph node was present in the left upper internal deep cervical region (Figure 3A), and this was also reduced to 10 × 10 mm after the second course (Figure 3B).

### 3.3. Clinical Assessment after Neoadjuvant Chemotherapy and the Definitive Treatment Decision

The gross response of the primary tumor and cervical lymph nodes was confirmed. After only two session of combination cisplatin and 5-FU neoadjuvant chemotherapy, the patient was judged to have achieved more than a partial response. After the patient provided informed consent for definitive surgical treatment, he underwent left enlarged tonsillectomy and left neck dissection under general anesthesia. His postoperative course was uneventful and he was discharged after 1 week.

After that, our oncology team subjected the specimens taken before neoadjuvant chemotherapy and after the second session to detailed analysis.

### 3.4. Histopathological Analysis before and after Neoadjuvant Chemotherapy

**Figure 4:**
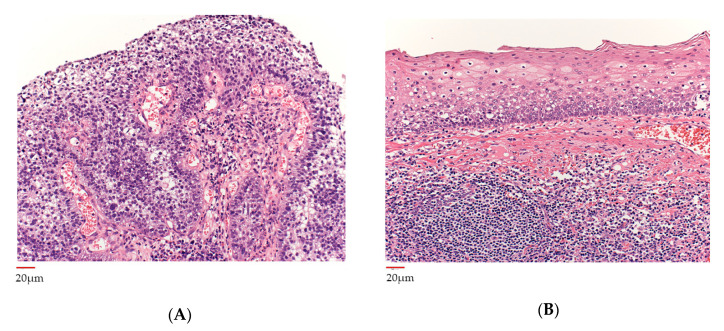
Histopathological findings of the primary tumor. (**A**) Biopsy specimen of primary tumor before neoadjuvant chemotherapy showing papillary proliferation in squamous epithelium around vascular connective tissue and nuclear atypia, diagnosed as squamous cell carcinoma. There is no obvious stromal infiltration. The subepithelium shows inflammatory cell infiltration. (**B**) Surgical specimen of the primary tumor after neoadjuvant chemotherapy showing no obvious cancer cells, preservation of the basement membrane, and no viable tumor remaining.

The pre-chemotherapy biopsy specimen showed the squamous epithelium with nuclear atypia proliferating in a papillary fashion with a core of vascular connective tissue. There was no obvious stromal infiltration and the subepithelium showed inflammatory cell infiltration. This was diagnosed as differentiated squamous cell carcinoma (Figure 4A). The post-chemotherapy specimen, no cancer cells were detected including nonviable cancer cells (Figure 4B).

### 3.5. p16 Analysis before and after Neoadjuvant Chemotherapy

**Figure 5:**
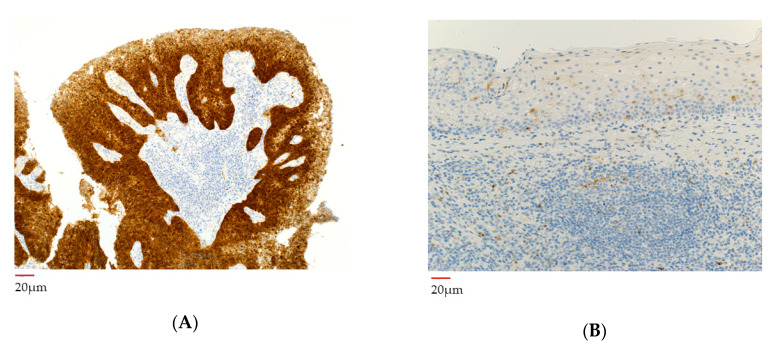
The findings of immunohistochemical staining for p16 in primary tumor. (**A**) Immunohistochemical staining for p16 in the biopsy specimen of the primary tumor before neoadjuvant chemotherapy showing diffusely strongly positive staining in the nuclei of > 70% of tumor cells, characteristic of HPV-positive squamous cell carcinoma. (**B**) Surgical specimen of the primary tumor after neoadjuvant chemotherapy showing no obvious neoplastic lesions and no p16-positive cells.

For immunostaining, p16 mouse monoclonal antibody (clone E6H4; ready-to-use) was used and then evaluated with the CINtec^®^ Histology Kit (Ventana Medical Systems, Inc., Oro Valley, AZ, USA). Immunohistochemical staining of the pre-chemotherapy specimen showed diffuse and strong staining in the nuclei of almost all tumor cells (Figure 5A). In the post-chemotherapy specimen, there was no obvious p16-positive cells indicating residual cancer cells (Figure 5B).

### 3.6. HPV Gene Integrated Cells before and after Neoadjuvant Chemotherapy

To confirm the presence or absence of high-risk viruses at the RNA level, RNA in situ hybridization was performed using the RNAscope^®^ assay to compare changes in high-risk HPV types before and after chemotherapy. RNA in situ hybridization was done as follows.

Briefly, RNAscope^®^ 2.5 HD Reagents Kit-BROWN assay kit and the HPV probe cocktail (Advanced Cell Diagnostic Inc, Newark, CA, USA) were used to detect high-risk HPV mRNA by RNA-ISH. RNAscope^®^ for high-risk HPV in this study covered type 16, 18, 26, 31, 33, 35, 39, 45, 51, 52, 53, 56, 58, 59, 66, 68, 73 and 82. Samples were deparaffinized, then activated by boiling, followed by protease digestion using a hybEZ™ oven. Hybridization was performed by incubating slides with the positive control, negative control and high-risk HPV reagent in the oven, then signal amplification was conducted Samples were encapsulated with xylene after H&E staining and DAB coloring. High-risk HPV expression levels were checked pre-chemotherapy specimen and also post-chemotherapy specimen.

**Figure 6:**
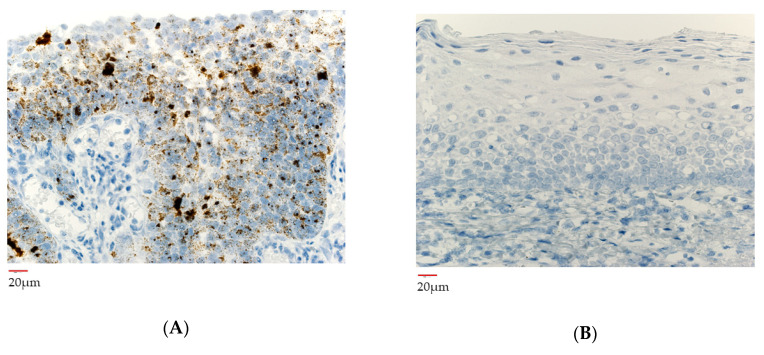
The findings of high-risk HPV viral antigens in RNA in situ hybridization. (**A**) Biopsy specimen of the primary tumor before neoadjuvant chemotherapy showing expression of high-risk HPV genotypes. (**B**) Surgical specimen of the primary tumor after neoadjuvant chemotherapy showing no signals of high-risk HPV genotypes.

Signals for high-risk HPV genotypes were detected in the pre-chemotherapy biopsy section of the primary tumor (Figure 6A). In contrast no those of signals were detected in the post-surgical specimen (Figure 6B). About for low-risk HPV genotypes, there were no positive signals both biopsy and the surgical specimen. There were also no positive signals for high-risk HPV genotypes in the dissected lymph nodes.

## 4. Discussion

In this case of p16-positive oropharyngeal carcinoma, pathological CR was confirmed in surgical specimens after two courses of neoadjuvant chemotherapy. A recent study reported a CR rate of around 50% for both the primary tumor and metastatic cervical lymph nodes after 3 courses of induction chemotherapy (cisplatin and docetaxel combination) in cases of p16-positive oropharyngeal carcinoma [13]. However, that study focused on the role of CT and MRI imaging in predicting pathological response, and there have been no reports on detailed pathological status. In this respect, the present case highlights the chemotherapy effect from the oncological viewpoint. Moreover, this is the first report of virological investigations in oropharyngeal cancer and of pathological CR after chemotherapy alone.

p16 has been reported as a surrogate marker for high-risk HPV related cancer [14]. In the present case, we confirmed the actual HPV status at the RNA level using RNAscope^®^. In this case, chemotherapy eliminated not only the pathological CR but also the positive signal of high-risk HPV. Considering the fact that this patient was an HPV carcinogenesis patient, the absence of high-risk HPV signal by chemotherapy alone may suggest that the risk of recurrence is even lower. Further accumulation of similar cases may support forgoing radiation therapy and perhaps even surgery might be unnecessary when such a response is achieved after neoadjuvant chemotherapy if close monitoring is possible.

In terms of the mechanism of virological response to chemotherapy, the direct antiviral effect of cisplatin on reovirus, vaccinia virus, HSV-1, and VZV in basic research has been reported [15], and a similar effect on papillomaviruses is expected. However, because the direct antiviral effects on different DNA viruses need to be studied individually, the antiviral effect against papillomaviruses is not clear at this time and future research is warranted. Exposure of HPV-infected cells to cisplatin may induce apoptosis, which may also have an antiviral effect. Although the oncogenic mechanism of HPV is attributed to the expression of E6 and E7 genes by high-risk HPV [16], cisplatin suppresses their expression and induces p53-induced apoptosis in HPV-infected cells [17].

Another possible mechanism for virological response to chemotherapy is the tumor immunopotentiation effect of the drug, which is not clear at this time. HPV-positive oropharyngeal lateral wall squamous cell carcinoma has an abundance of tumor-infiltrating CD8^+^ and Foxp3^+^ T cells compared to negative ones [18]. HPV-positive oropharyngeal carcinoma has also been presented as a potential benefit of immunotherapy, but it is still in the research stage. In addition, an increase in tumor-infiltrating lymphocytes in head and neck squamous cell carcinoma tissues after neoadjuvant chemotherapy including cisplatin and 5-FU has recently been reported [19], and the tumor immunopotentiation effect of these agents may be possible. However, in this case, although inflammatory cell infiltration was strong in the pre-chemotherapy specimen, only a few tumor-infiltrating lymphocytes were found, suggesting that the virus was likely cleared as a result of the virus-killing effect of the drugs used, or by an unknown mechanism. 

In the case that viral RNA is observed in pathological CR cases after neoadjuvant chemotherapy, we should consider additional treatment from a viewpoint of the residual risk of recurrence. Adjuvant administration of an antiviral drug or therapeutic HPV vaccine might be effective. Therapeutic HPV vaccines targeting E6 and E7 have been studied in cervical cancer caused by HPV16 infection. In addition, using RNA interference to inhibit E6 and E7 genes for the suppression of cervical cancer has been investigated [20,21] and this may become a common therapeutic approach for p16-positive oropharyngeal carcinoma and other HPV-related cancers in the future.

Neoadjuvant chemotherapy may also help prevent distant metastasis. In one study, 10% of p16-positive oropharyngeal cancer patients treated with radiation (with chemotherapy) were failed to be cured by distant metastases alone, and they account for almost 50% of all failed cures [22]. This suggests that chemotherapy prior to definitive therapy is reasonable and also that chemotherapy alone as a systemic treatment option may be an option. Moreover, low-dose chemotherapy or continued use of E6 and E7 gene suppressors may be considered in older patients or patients with renal dysfunction. If more such data are accumulated in the future, chemotherapy for head and neck cancer may become a more focused treatment strategy.

Finally, tumor immunotherapy might be the focus of head and neck cancer treatment into the future. As mentioned above, we also present the possibility that cancer immunotherapy may also be effective. The findings in our case suggest the possibility of a new treatment strategy for oropharyngeal carcinoma, such as specific tumor immunotherapy for HPV in cases of pathological CR after induction chemotherapy when the HPV virus persists. Further studies are needed to explore this possibility.

In conclusion, it is known that p16-positive cases have better prognosis than p-16 negative cases [23] and studies are underway to try to reduce the dose intensity of chemoradiotherapy [24,25]. The CR achieved after neoadjuvant chemotherapy in our case supports the evidence that conventional high intensity chemoradiotherapy might be overly invasive in p16-positive oropharyngeal carcinoma. In addition, based on the pathological responses to neoadjuvant chemotherapy seen in this case, neoadjuvant chemotherapy may offer an alternative to reduce the intensity of radiation therapy or even forgo it, and instead continue with chemotherapy, implement a vaccine intervention, or start tumor immunotherapy. Optimal personalized medicine that includes the options of chemotherapy, radiation therapy, surgery, immunotherapy, and vaccination should be provided to help achieve a complete cure in p16-positive cases.
